# Lung Edema Clearance: Relevance to Patients with Lung Injury

**DOI:** 10.5041/RMMJ.10210

**Published:** 2015-07-30

**Authors:** Zaher S. Azzam, Jacob I. Sznajder

**Affiliations:** 1 Internal Medicine “B”, Rambam Health Care Campus, Department of Physiology and Biophysics, The Rappaport Family Faculty of Medicine and Research Institute, Technion, Israel Institute of Technology, Haifa, Israel; 2 Division of Pulmonary and Critical Care Medicine, Northwestern University, Chicago, IL, USA.

**Keywords:** Acute lung injury, alveolar epithelium, alveolar fluid clearance, Na,K-ATPase, pulmonary edema

## Abstract

Pulmonary edema clearance is necessary for patients with lung injury to recover and survive. The mechanisms regulating edema clearance from the lungs are distinct from the factors contributing edema formation during injury. Edema clearance is effected via vectorial transport of Na^+^ out of the airspaces which generates an osmotic gradient causing water to follow the gradient out of the cells. This Na^+^ transport across the alveolar epithelium is mostly effected via apical Na^+^ and chloride channels and basolateral Na,K-ATPase. The Na,K-ATPase pumps Na^+^ out of the cell and K^+^ into the cell against their respective gradients in an ATP-consuming reaction. Two mechanisms contribute to the regulation of the Na,K-ATPase activity:recruitment of its subunits from intracellular compartments into the basolateral membrane, and transcriptional/translational regulation. Na,K-ATPase activity and edema clearance are increased by catecholamines, aldosterone, vasopressin, overexpression of the pump genes, and others. During lung injury, mechanisms regulating edema clearance are inhibited by yet unclear pathways. Better understanding of the mechanisms that regulate pulmonary edema clearance may lead to therapeutic interventions that counterbalance the inhibition of edema clearance during lung injury and improve the lungs’ ability to clear fluid, which is crucial for patient survival.

## INTRODUCTION

Pulmonary edema is a life-threatening condition of fluid excess in the lungs that causes impaired gas exchange with consequent symptoms that range from mild shortness of breath to acute respiratory failure. Approximately 56% of intensive care unit patients suffer from acute respiratory failure (ARF), with one-third of those subsequently dying.^1^ The pathogenesis of ARF can be classified to cardiogenic and non-cardiogenic pulmonary edema. Acute heart failure is the most common cause of increased hydrostatic pressure and is very prevalent, with almost 658,000 emergency department visits in the United States per year. The mortality rate from acute cardiogenic pulmonary edema ranges from 12% to 15%.^2^ In the USA, overall costs of heart failure in 2010 have been estimated at $39.2 billion, with hospitalization representing approximately 80% of direct treatment costs for heart failure.^3^

Acute lung injury (ALI) that is due to increased permeability pulmonary edema is also common, with an incidence of 86 per 100,000 person-years, and equates to over 190,000 cases and 74,500 fatalities annually in the United States. Although mortality has declined, recent studies still report an approximate 25% death rate.^2^

For many years, it was believed that fluid accumulation in the lung depends only on the abrogation of balanced Starling forces—the hydrostatic pressure and oncotic pressures.^4^,^5^ However, more recently it has been demonstrated that the alveolar epithelium has an active role in clearing edema out of the alveoli, a process called alveolar fluid clearance (AFC).

## LUNG STRUCTURE

The lung is responsible for gas exchange: enriching the circulation with oxygen (O_2_) and extruding carbon dioxide (CO_2_). The structure of the lungs is designed to facilitate gas exchange by enabling the transit of gases through the respiratory airways, and, as the gases reach alveolar sacs and alveolus clusters, gas exchange actually occurs. The alveoli are tightly wrapped with blood vessels allowing the diffusion of oxygen from the alveoli to the blood-stream of the alveolar blood vessels. Then, oxygenated blood is perfused throughout the body where gas exchange occurs in the capillary beds.^6^

**Figure 1:**
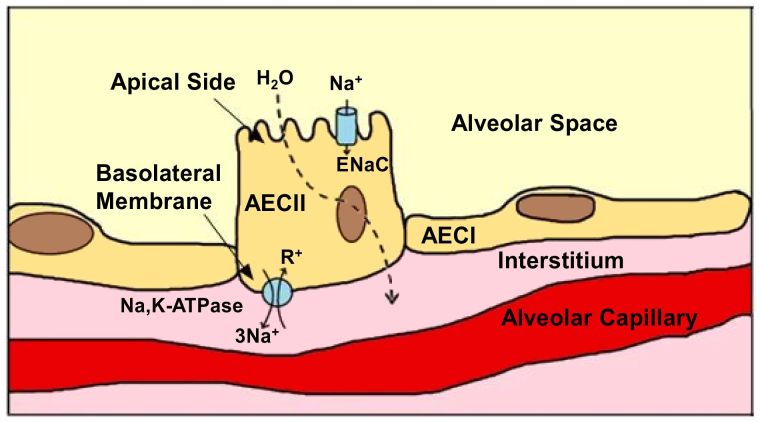
Schematic Representation of Alveolar Epithelial Cells with the Components that Contribute to the Alveolar Fluid Clearance Process AECI, alveolar epithelial cell type I; AECII, alveolar epithelial cell type II; ENaC, epithelial Na^+^ channel; Na,K-ATPase, sodium-potassium pump.

Since gas exchange relies on diffusion, it is crucial that the layer separating the alveolar space from the interstitium is thin and permeable.^6^ To ensure this environment, alveoli are built of a monolayer epithelium that contains two alveolar epithelial cells (Figure 1), types I and II (AECI and AECII, respectively), and macrophages.^7^ Moreover, the alveolar space must be free of fluids and open. *In utero*, the fetal lung is filled with fluid that is removed shortly after birth, mainly because active reabsorption of sodium ions (Na^+^) across the alveolar epithelium creates an osmotic force favoring reabsorption of alveolar fluid.^8^

Alveolar epithelial cells type I are squamous with a diameter of about 50–100 μm; however, they are very thin, thus minimizing the diffusion distance between the alveolar airspace and the pulmonary capillaries, which facilitates gas exchange. Although they constitute 5%–10% of all lung cells,^9^ AECI cover more than 90% of the alveolar surface as they are very large and have thin cytoplasmic extensions. Recently, it was reported that AECI play an active role in water permeability and the regulation of alveolar fluid homeostasis.^10^,^11^

The alveolar epithelial cells type II are smaller and cuboidal, with a diameter of 21 μm in rats and 50 μm in humans. They occupy only ~5% of the surface area, yet AECII constitute ~15% of all lung cells and 60% of alveolar epithelial cells. They produce, secrete, and recycle lung surfactant; they transport ions, participate in lung immune responses, and can also be converted to AECI to repair damaged epithelium or during fetal lung development. The AECII has a distinct morphology with characteristic lamellar bodies and a bipolar plasma membrane, consisting of an apical side that has short microvilli and a basolateral domain. These cells contain a wide range of transport proteins, including epithelial Na^+^ channels (ENaCs), Na,K-ATPase, Na-H exchanger (NHE), and aquaporin-3 (AQP3).^12^–^16^

## HISTORIC PERSPECTIVE

Normand et al. demonstrated that fetal lamb lungs can absorb fluid from the airspaces at birth,^17^ and by using ^131^I tracer Walters and Olver calculated the rate of lung liquid secretion in fetal lambs.^18^ Matthay et al. reported that mature sheep lungs have the ability to clear edema.^19^ Since then, alveolar fluid clearance has been extensively investigated.^4^,^20^–^25^

## ALVEOLAR ACTIVE SODIUM TRANSPORT MECHANISM

Alveolar fluid clearance (AFC) is an active process carried out mostly by the apical epithelial Na^+^ channels, and the basolateral Na,K-ATPase is involved in AFC.^13^,^24^

Briefly, Na^+^ enters the alveolar epithelial cells through the apical amiloride-sensitive Na^+^ channels (ENaC), is transported through alveolar epithelial cells, and by a process that consumes energy is pumped out of the cell by the Na,K-ATPase located in the basolateral membrane in exchange for potassium entry in a ratio of 3:2 Na^+^–K^+^ against their chemical gradient. This active vectorial Na^+^ flux produces a transepithelial osmotic gradient that causes water to move from the airspaces following the gradient.^8^,^26^–^28^

**Figure 2:**
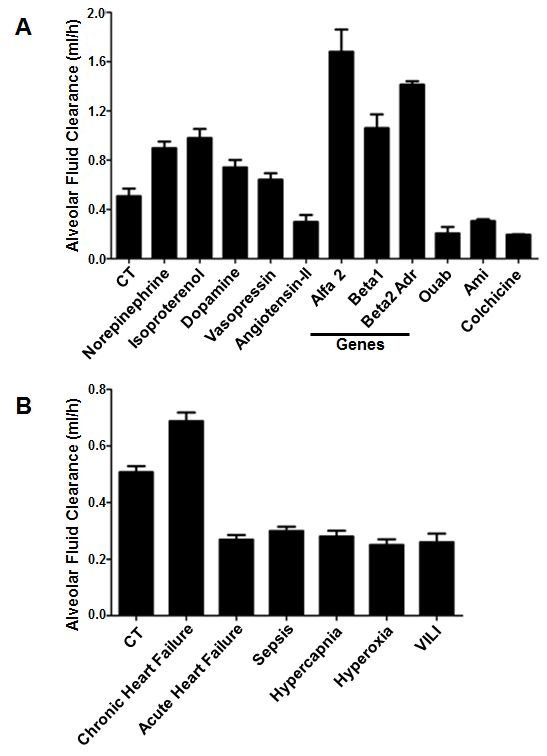
The Effect of Various Pharmacologic and Pathophysiologic Conditions on Alveolar Fluid Clearance **A:** The rate of alveolar fluid clearance (AFC) was modulated following therapeutic interventions: catecholamines, vasopressin, and gene therapy upregulated AFC; however, the administration of endothelin, angiotensin, amiloride, ouabain, or colchicine inhibited active sodium transport and thus AFC. The data were adapted from references 13, 27, 28, 31, 33, 36, 37. **B:** Alveolar fluid clearance (AFC) was decreased in the various states of acute lung injury, such as sepsis, hyperoxia, hypercapnia, and ventilation-induced lung injury. Moreover, in rats exposed to acutely increased left atrial pressure (e.g. acute left heart failure) AFC was inhibited; whereas AFC was significantly upregulated in chronic heart failure rats. The data were adapted from references 34, 40–43. The bars represent mean ± SEM. Alfa2, α2-subunit of Na,K-ATPase; Ami, amiloride; beta1, β1-subunit of Na,K-ATPase; beta2 adr, β2 adrenergic receptor; CT, control; Ouab, ouabain; VILI, ventilation-induced lung injury.

Alveolar fluid reabsorption can be modulated by pharmacologic agents, gene therapy, and other interventions. Catecholamines, growth factors, vasopressin, aldosterone, overexpression of Na,K-ATPase subunits and chronic heart failure model increase alveolar fluid reabsorption (Figure 2A).^16^,^29^–^40^

Active Na^+^ transport and edema clearance are inhibited by interventions that can be divided into several categories:

1. General AFC inhibitors such as the sodium channel blocker, amiloride,^41^ and the Na,K-ATPase inhibitor, ouabain.^5^
2. Consequences of acute lung injury (ALI), hypoxia,^42^ and hypercapnia that impair the alveolar epithelial function by increasing intracellular calcium levels.^43^,^44^
3. Mechanisms of ALI including sepsis,^45^ hyperoxia,^46^ high tidal volume ventilation and ventilation-induced lung injury,^47^ acute left atrial hypertension,^48^ andendothelin (Figure 2).^49^

Notably, aerosolized or intravenous β_2_-agonist therapy did not improve clinical outcomes in patients with lung injury; therefore, the use of β_2_-agonist therapy was not recommended in mechanically ventilated patients with lung injury.^50^,^51^ A more recent study appears to shed light onto the disparate effect of β_2_-agonist therapy *in vitro* in animals, and in patients with lung injury. Apparently, the β_2_-adrenergic receptor (β_2_AR) on alveolar macrophages can augment the release of IL-6, thus linking the sympathetic nervous system, by β2AR signaling, with lung inflammation and enhanced susceptibility to thrombotic cardiovascular events, which could have negative effects on the outcome of patients with acute lung injury.^52^

Alveolar fluid clearance reflects active sodium transport, and several mediators participate in this process, including Na,K-ATPase, Na^+^ channels, aquaporins, and others. The sodium–potassium pump, an energy-consuming enzyme (Na,K-ATPase), is a heterodimeric integral membrane protein that is synthesized in polysomes related to the rough endoplasmic reticulum. It is composed of two subunits: an α-subunit (a catalytic 110-kDa unit) and a β-subunit (a regulatory 55-kDa unit). The α-subunit contains binding sites for ATP hydrolysis, Na^+^, K^+^, and cardiac glycosides. There are at least three isoforms of α (α1, α2, and α3); they differ by their affinity to sodium, ouabain, and tissue distribution. The β-subunit is thought to be responsible for incorporating the α-subunit into the plasma membrane; there are three known isoforms of β-subunit.

**Figure 3:**
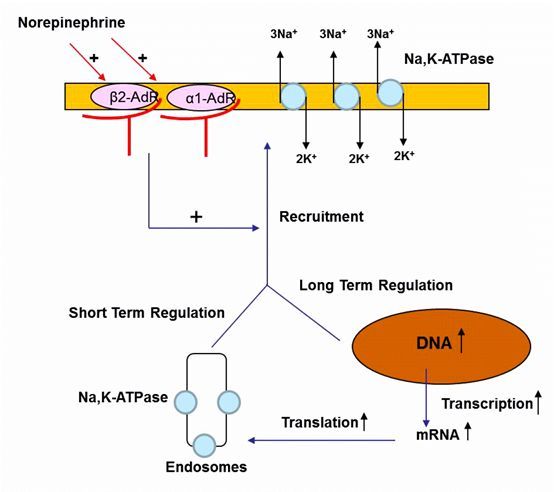
Schematic Representation of Active Sodium Transport in the Alveolar Epithelial Cell Depicting Apical Na^+^ Channels, Basolaterally Located Na,K-ATPase, Aquaporins, and Co-transporters Sodium enters through the apical membrane via Na^+^ channels and is extruded by the Na,K-ATPase, with water following iso-osmotically. Proposed mechanisms by which norepinephrine upregulates active sodium transport and alveolar clearance.

A critical role is played by Na,K-ATPase in the homeostasis of Na^+^ and K^+^ during altered salt intake and pH regulation, besides its other important functions in several organs; therefore it is strictly regulated.^8^,^53^ The Na,K-ATPase can be regulated at the level of expression, internalization, and recruitment of the pump proteins to the basolateral membrane (Figure 3). For example, catecholamines enhance the ability of the lungs to clear edema by recruiting Na,K-ATPase from the alveolar epithelial cell (AEC) cytosol to the basolateral membranes within minutes and thus increasing the activity of the pump. This process is mediated via the cAMP/PKA pathway.^30^,^54^,^55^ In contrast, the adverse effects on AFC of hypoxia, hypercapnia, sepsis, and endothelin are due to the AMPK/PKC pathway, in which Na,K-ATPase is phosphorylated, leading to its endocytosis.^42^,^45^,^49^,^56^

An important contributor to alveolar sodium transport is the sodium channel (ENaC). The ENaC is a heterotrimeric protein that can be composed by different combinations of three known subunits: αENaC, βENaC, and γENaC. Alveolar epithelial cells contain three types of channels with different selectivity properties: (1) a Ca^2+^-activated non-selective Na^+^ channel (NSC) composed of α-subunits alone, (2) a Na^+^-selective (moderately selective) channel composed of a combination of αENaC and βENaC or γENaC, and (3) the highly NSC composed of the three different subunits. The ENaC is located on the apical portion of AECs and plays a crucial role in sodium transport and AFC.^28^,^30^,^57^ Moreover, it has been shown that knocking out αENaC leads to defective AFC and premature death in newborn mice.

Water channels or aquaporins (AQPs) are expressed in the lungs. AQP1 is located in both the apical and basolateral aspects of endothelial cells and fibroblasts, and AQP3, AQP4, and AQP5 are expressed in the respiratory epithelium. In the human respiratory tract, AQP5 is expressed in the apical surface of AECI and AQP3 in the apical and basal membrane of AECII. Targeted deletions of AQPs in transgenic mice suggest that AQPs are not essential for alveolar fluid clearance; however, other compensatory mechanisms could have taken place instead of the deletion of AQPs.^58^

While the contribution of AECI to alveolar fluid clearance has been suggested, further studies to evaluate the role of distal epithelial cells are necessary. We also need to explore the role of chloride channels and their regulation, particularly in the pathways of activation as well as functional contributions of AQPs and cystic fibrosis transmembrane conductance regulator (CFTR). Additionally, signal transduction pathways as well as translation and post-translational pathways need to be studied as they may inform the field and help with novel therapies to modulate these pathways to enhance pulmonary edema clearance in patients with lung injury.

## CONCLUSIONS

The mechanisms of lung edema clearance contrast with the regulation of pulmonary edema formation. Clearance of edema fluid is an active process that requires active transport of Na^+^ out of alveolar airspaces, with water following the osmotic gradient. The regulation of vectorial Na^+^ transport across the alveolo-capillary barrier is mediated mostly by apical Na^+^ channels and basolaterally expressed Na,K-ATPases. In patients with acute respiratory distress syndrome and lung injury the mechanisms regulating alveolar fluid reabsorption are impaired, and the restoration of the alveolar epithelial function to keep the lungs dry is important for normal gas exchange to occur and patients to survive. More knowledge about the mechanisms regulating lung edema clearance is needed in order to develop novel therapeutic strategies to accelerate fluid clearance and improve the alveolar epithelial function.

## Abbreviations

AEC: alveolar epithelial cell
AFC: alveolar fluid clearance
AQP: aquaporin
β2AR: β2-adrenergic receptor
CFTR: cystic fibrosis transmembrane conductance regulator
ENaC: epithelial Na^+^ channel
NHE: Na-H exchanger
NSC: non-selective Na^+^ channel.
